# The impact of nutritional status on health-related quality of life in hemodialysis patients

**DOI:** 10.1038/s41598-022-07055-0

**Published:** 2022-02-22

**Authors:** Lucia Visiedo, Laura Rey, Francisco Rivas, Francisca López, Begoña Tortajada, Rafael Giménez, Jimena Abilés

**Affiliations:** 1 Pharmacy and Nutrition Unit of the Costa del Sol Healthcare Agency, Marbella, Spain; 2 Research and Innovation Unit of the Costa del Sol Healthcare Agency, Marbella, Spain; 3 Nephrology Unit of the Costa del Sol Healthcare Agency, Marbella, Spain; 4 Department of Nutrition and Bromatology, University of Granada, Granada, Spain

**Keywords:** Kidney diseases, Renal replacement therapy, Nutrition

## Abstract

Malnutrition is frequent in hemodialysis (HD) patients. Nutritional deficiencies may negatively impact quality of life (QOL). This study examines the utility of the Malnutrition-Inflammation Score (MIS) in detecting nutritional risk (NR) and assesses the correlation between nutritional status and QOL in dialysis patients upon starting a nutritional intervention program (NIP). One hundred and twenty patients were included in this cross-sectional study. The MIS was used to detect NR and the Kidney Disease Quality of Life (KDQOL-SF) instrument version 1.2 was used to assess QOL. 62% of patients were found to be at NR (MIS > 5). Nutritional status was significantly correlated with all generic QOL sub-scales. On a multiple linear regression analysis, malnutrition showed the highest level of explanation in the Kidney Disease Summary Component which explained 28.9% of the variance; the Physical Component Summary which explained 33% of the variance; and the Mental Component Summary which explained 21.5% of the variance. Malnutrition was found to be the most significant predictor of impaired scores on the KDQOL-SF. The use of MIS to identify patients at NR and a nutritional assessment to detect malnutrition in its early stages are important given the effects a NIP can have on improving QOL in HD patients.

## Introduction

Malnutrition is frequent among patients with end-stage kidney disease receiving hemodialysis (HD). Its prevalence in HD patients ranges from 18 to 75%^1^. Maintenance HD entails a risk of malnutrition due to the catabolic effects of this renal replacement therapy, inadequate dietary intake due to poor appetite caused by the uremic environment and dietary restrictions, loss of nutrients through the dialysis membrane, inflammation, and metabolic acidosis, which can lead to protein energy wasting (PEW) syndrome^2,3^. PEW is a term related to cachexia, malnutrition, and inflammation proposed by the International Society of Renal Nutrition and Metabolism (ISRNM) for the multiple nutritional and catabolic alterations that occur in chronic kidney disease (CKD) and it is associated with morbidity and mortality^4^

Malnutrition is associated with an increase in morbidity, a decrease in functional capacity, and a greater number and duration of hospital admissions, all of which may cause a low health-related quality of life (QOL) and impact patients’ emotional, physical, and psychosocial health. It has been described that malnourished patients have a worse QOL and thus, the early diagnosis and treatment of malnutrition is important^5,6^. Although nutritional status has been shown to impact QOL in HD patients, there is a limited body of evidence supporting this relationship.

In 2017, the Pharmacy and Nutrition and Nephrology Department at our hospital designed and implemented a nutritional care model for HD patients to enable the early detection of nutritional risk (NR), facilitate periodic nutritional assessment and monitoring, and implement a nutritional intervention program (NIP) at early stages prior to the onset of malnutrition so as to prevent further deterioration in QOL.

This study aims to describe the relationship between nutritional status and QOL among maintenance hemodialysis patients prior to the implementation of a NIP. The Malnutrition-Inflammation Score (MIS) tool was used to detect NR and Kidney Disease Quality of Life (KDQOL-SF) instrument version 1.2 to assess QoL.

## Materials and methods

### Study design

The sample size required in order to evaluate differences in perceived health-related quality of life based on the presence of malnutrition was calculated using a normalized difference of means of 0.6, a 95% confidence level, a minimum power of 80%, and a 1:1 ratio of presence or absence of malnutrition. Based on these premises, 88 patients would need to be evaluated. To minimize possible losses, this number was increased by 20%. Therefore, the minimum sample size was 106 patients.

In this descriptive study, the nutritional care model for HD patients began with a complete nutritional assessment of food intake and symptoms that could affect nutritional status and QOL, in accordance with the clinical guidelines of The National Kidney Foundation’s Kidney Disease Outcomes Quality Initiative on nutritional support for HD patients^7^.

As per our hospital’s protocol, the MIS questionnaire is administered every three months and the KDQOL-SF is administered twice a year. Both are performed at the beginning of HD sessions by nutritionists and trained nursing assistants who work in the Nephrology Unit. Blood samples are taken at the beginning of the HD session.

Ethical approval was granted by Costa del Sol Research Ethics Committee on May 30, 2020 with approval number 85-05-2019. The ethical principles set forth in the most recent version of the Declaration of Helsinki and the standards of good clinical practice were adhered to. All participants signed an informed consent form prior to their inclusion in the study.

### Study population

**Figure 1:**
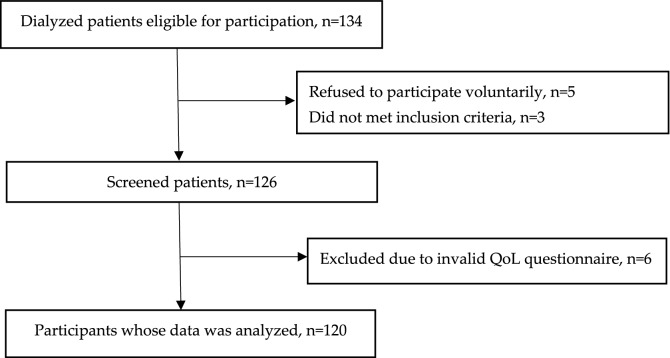
Flowchart of participants included in the study.

Participants met the following inclusion criteria: adults (18 years or older) who had not previously consulted with a dietician and who had been in the HD program for at least three months. HD sessions were held three times per week for four hours. A flowchart describing the inclusion of participants in this study is shown in Fig. 1. One hundred and twenty-six patients underwent an initial assessment. Of them, 120 patients had a valid QoL assessment, which is the sample used for this study. No participants dropped out of the study.

#### Malnutrition-inflammation score

The MIS was used to determine NR and a nutritional assessment was subsequently used to establish a nutritional diagnosis. Following its calculation, patients were categorized as well-nourished or malnourished. Malnourished patients were then classified as having mild, moderate, or severe protein-energy malnutrition or protein malnutrition.

Although several methods have been used to assess nutritional status in HD patients, there is no gold standard technique. The MIS, described by Kalantar et al.^8^, uses components of the conventional Subjective Global Assessment (SGA)^9^ and also includes comorbidity according to time on HD as well as biochemical parameters such as albumin, total iron-binding capacity, and transferrin. The MIS has four sections: nutritional history, physical examination, body mass index, and laboratory values. Total scores range from 0 to 30 points and scores > 5 indicate the presence of NR. The MIS is widely used in CKD patients^10,11^, is supported by studies which have demonstrated its value as a predictor of mortality and morbidity in HD patients^12–14^, and is a useful tool for detecting PEW in CKD patients^15^.

##### Nutritional assessment

A nutritional assessment is a dynamic process that draws on several parameters that allow for an initial assessment. These parameters include the medical record, dietary record, and a physical examination. The medical record includes all data regarding the patient’s health status throughout his or her life and is focused on aspects that may increase risk of malnutrition. The dietary record includes data related to the patient’s dietary habits in order to identify problems that may have an adverse effect on their health. For this parameter, we used a food diary (including hydration) for three days, one of which was a weekend day. The caloric and nutritional value of the diet was quantified using DIAL software^16^. Energy, protein, potassium, and phosphorus requirements were calculated according to the recommendations of the KDOQI guidelines^7^. For the rest of nutrients, the food composition tables for the Spanish population were considered^17^ in order to calculate compliance with the proposed nutritional objectives. A physical examination is necessary in order to determine changes in weight.

After gathering these three items, we established a nutritional diagnosis according to the consensus of the malnutrition established by the Spanish Society of Enteral and Parenteral Nutrition and the Spanish Society of Medical Documentation^18^. This codification classifies malnutrition into three groups: protein malnutrition, caloric malnutrition, or mixed malnutrition (caloric-protein). This last classification is divided into different degrees according to the severity of malnutrition: mild, moderate, or severe.

#### Quality of life

QOL was measured using validated Spanish version of the KDQOL-SF version 1.2^19^. It includes 43 specific items for patients with kidney disease organized into 11 specific dimensions of the disease. They include symptom/problem list, effects of kidney disease, burden of kidney disease, work status, cognitive function, quality of social interaction, sexual function, sleep, social support, dialysis staff encouragement, and patient satisfaction. All of these aforementioned items form part of the kidney disease summary component (KDSC). Furthermore, the KDQOL-SF also includes a section with the 36 generic items of the SF-36 questionnaire. It is organized into eight dimensions and two summary scores: the physical component summary (PCS) and the mental component summary (MCS) scores. Items include physical functioning, role-physical, pain, general health, emotional well-being, role-emotional, social function, and energy/fatigue.

Each question is numerically coded and then scored on a scale of 0 to 100; higher values reflect better QOL. It also includes an item about health measured on a scale of 0–10, where 0 indicates "worst possible health (as bad as or worse than being dead)" and 10 indicates "best possible health."

#### Physical function

The Barthel index evaluates performance in activities of daily living^20^. The total score possible ranges from 0 to 100, with lower scores suggesting increased disability.

The Downton scale index was used to assess the risk of falls. This instrument consists of five modules: previous falls, medication, sensory deficits, mental state and gait, reporting 11 risk factors, which are summarized into a score ranging from 0 to 11. Scores ≥ 3 points indicate a significantly increased risk^21^.

#### Statistical methods

Data are presented as means ± standard deviation. Categorical variables are shown as percentages. The Pearson correlation coefficient was used for independent quantitative variables, the Mann–Whitney U test for dichotomous qualitative variables, and the ANOVA test for qualitative variables with three or more categories.

In order to explore how each sociodemographic and clinical characteristic influences QOL, a multiple linear regression analysis was performed. KDSC, PCS, and MCS were the outcome variables and backward stepwise selection was used for independent variables with an entry criterion of p < 0.05 and an exit criterion of p > 0.1. β-coefficients were calculated with the respective 95% confidence intervals.

The level of statistical significance was established as p < 0.05. All data were analyzed using the SPSS statistical software package for Windows, version 15.0 (SPSS Inc., Chicago, Ill., USA).

## Results

A total of 120 HD patients with a mean age of 68 ± 16 years were included. Diabetes mellitus (DM) was the most frequent etiology of kidney disease. Sixty-seven percent of participants were male. According to the Barthel Index, 53% were classified as dependent for activities of daily living. Other relevant sociodemographic and clinical characteristics are described in Table 1.

**Table 1:** Sociodemographic and clinical characteristics of the total population.

**Sociodemographic characteristics**			
Age, years (SD)	68 (16)	Living situation, n (%)	With a partner: 56 (46.7) Alone: 30 (25) With children: 18 (15.4)
Sex, n (%)	Male: 80 (67) Female: 40 (33)	Institutionalized, n (%)	4 (3.3)
Barthel Index, n (%)	64 (53) Dependent	Race, n (%)	Caucasian: 103 (85)
Downton Fall Risk Index (SD)	6 (1.5)	Time on dialysis, years (SD)	5.5 ± 3.6
Marital status, n (%)	Married: 82 (68) Single: 19 (16) Widowed: 16 (13)	Smokers, n (%)	45 (35.8)
Education level, n (%)	≤ Secondary school: 88 (73) > University 32 (27)	Employment status, n (%)	Employed: 29 (24) Not employed: 91 (76)
**Clinical characteristics**			
Cause of end-stage renal disease, n (%)	Diabetic nephropathy: 33 (27.5) Nephroangiosclerosis: 22 (18) Cardiovascular: 12 (10)	Weight, kg (SD)	70.5 (15.4)
Comorbidity, n (%)	DM: 55 (45) Arterial hypertension: 35 (30) COPD: 12 (10)	Body mass index, kg/m^2^ (SD)	26.1 (5.2)
Kt/V, (SD)	1.33 (0.3)		
**Biochemical characteristics**			
Hemoglobin, g/dL (SD)	10.8 (2.6)	Sodium, mEq/L (SD)	136.8 (12.2)
Creatinine, mg/dL (SD)	6.4 (2.5)	Potassium, mEq/L (SD)	5.1 (0.7)
Glomerular filtration rate, mL/min	8.6 (3.7)	Phosphorus, mg/dL (SD)	3.3 (0.5)
Urea, mg/dL (SD)	126.9 (38.2)	PTH, pg/mL (SD)	301.5 (193.8)
Urea post-dialysis, mg/dL (SD)	36 (18.3)	Glycated hemoglobin, % (SD)	7 (1.2)
Glucose, mg/dL (SD)	130 (55.3)	CRP, mg/L (SD)	13.9 (18)
Albumin, g/dL (SD)	3.3 (0.5)	Total protein, g/dL (SD)	6.2 (0.7)
Transferrin, mg/dL (SD)	171.6 (40.6)	Prealbumin, mg/dL (SD)	25.8 (9.7)
Cholesterol, mg/dL (SD)	143 (35.4)	Ferritin, mg/dL (SD)	94.6 (20.6)
Vitamin D, ng/mL (SD)	16 (11.3)		

Data are expressed as mean ± standard deviation for normal data.

*DM* Diabetes Mellitus, *COPD* chronic obstructive pulmonary disease, *CRP* C-reactive protein, *PTH* Parathyroid hormone.

**Figure 2:**
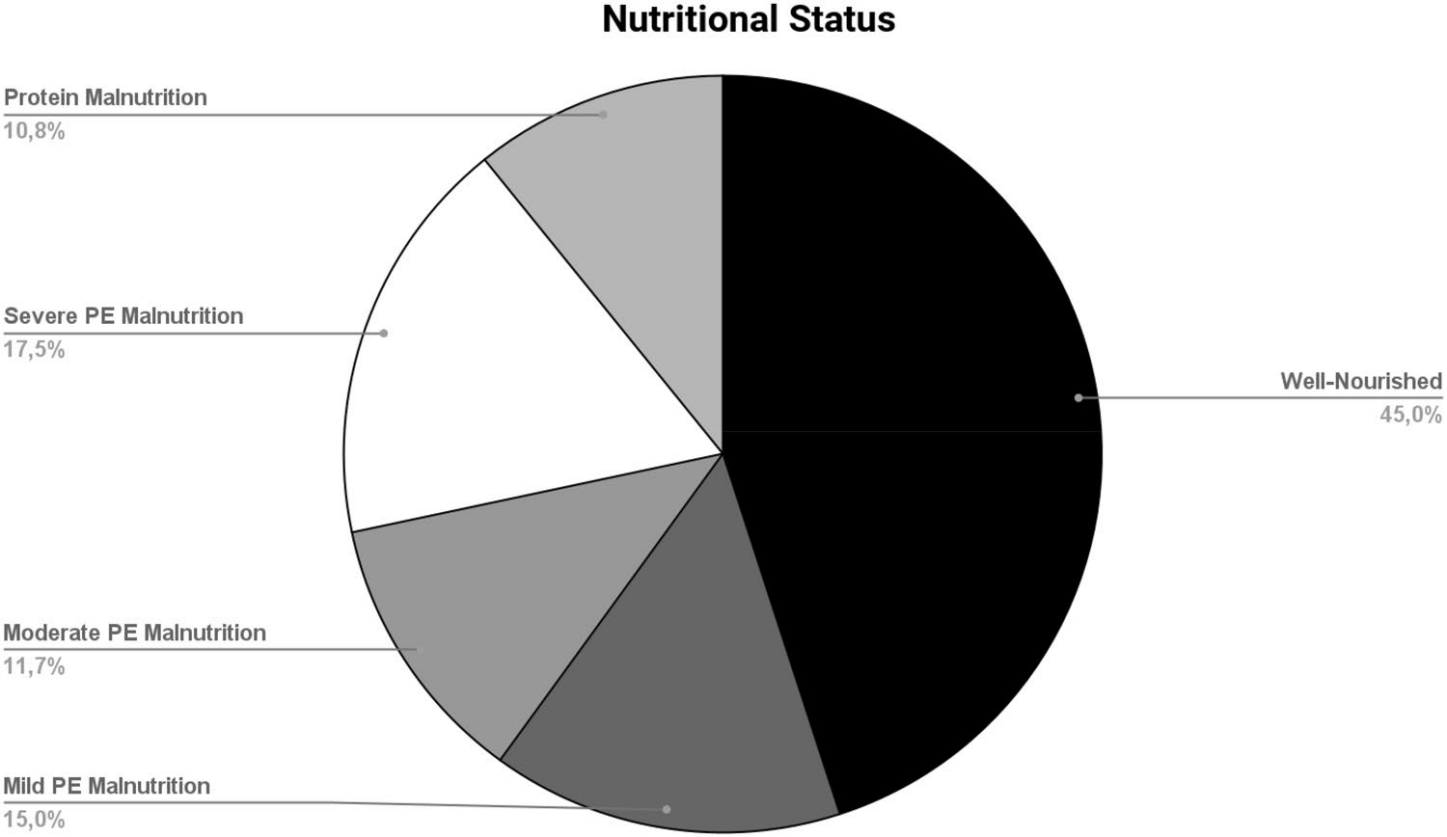
Nutritional status. Data are expressed as percentages. *PE* protein-energy.

The mean MIS scale score was 8.4 ± 3.5 and 62% of patients were found to be at NR (MIS > 5). After the screening, a nutritional assessment was conducted and malnutrition was detected in 55%. Of them, nearly 20% presented with severe protein-energy malnutrition (Fig. 2). No cases of caloric malnutrition were found.

All patients responded to the KDQOL-SF version 1.2 questionnaire while being monitored by trained staff.

The different dimensions were classified in two groups based on the total scores. The areas that had higher scores (> 80) were quality of social interaction, dialysis staff encouragement, and patient satisfaction with the care received. Lower scores (< 50) were found on effects of kidney disease, burden of kidney disease, sexual function, cognitive function, sleep, and work status. On the specific part, the mean KDSC score was 56 ± 10 points. On the generic part (SF-36), the area with the highest score was emotional well-being and those with lower scores were physical functioning, role-physical, energy/fatigue, and general health. The PCS and MCS had a total score < 50.

Fluid and dietary restrictions are two aspects that are bothersome in the daily life of HD patients and these aspects are inquired about in the effects of kidney disease dimension. Ninety percent of patients reported some degree of being bothered by fluid restrictions and 92% indicated being bothered by dietary restrictions; 25% and 20% of patients, respectively, indicated they were extremely bothered on these items.

The MIS and nutritional status were compared to scores on the KDQOL-SF components (Table 2). Patients who had an MIS score ≤ 5 and well-nourished patients had higher scores on the QOL final summary components (p < 0.001). In terms of the different sub-scales of this questionnaire, on the KDC, the areas that received significantly better scores among these patients were symptoms/problem list (p < 0.001), effects of kidney disease (p < 0.001), burden of kidney disease (p < 0.001) and sleep (p < 0.001). As for SF-36 components, all areas had significantly higher scores among patients who met these criteria (p < 0.001).

**Table 2:** Scores on the general summary areas according to MIS scale scores and nutritional status.

MIS score				Nutritional status		
	MIS ≤ 5 (n = 46)	MIS > 5 (n = 74)	p value	Well-nourished (n = 54)	Malnourished (n = 66)	p value
**Kidney disease components (SD)**						
Symptoms/problems	85 (11)	65 (20)	**< 0.001**	85 (11)	62 (19)	**< 0.001**
Effects of kidney disease	57 (21)	42 (20)	**< 0.001**	56 (22)	40 (19)	**< 0.001**
Burden of kidney disease	36 (19)	24 (23)	**< 0.001**	37 (20)	22 (22)	**< 0.001**
Work status	42 (34)	29 (24)	**0.022**	41 (33)	28 (24)	NS
Cognitive function	43 (39)	39 (27)	NS	42 (38)	39 (27)	NS
Quality of social interaction	41 (39)	39 (29)	NS	40 (38)	40 (29)	NS
Sexual function	66 (32)	53 (37)	**0.046**	63 (36)	53 (36)	NS
Sleep	52 (14)	41 (14)	**< 0.001**	51 (15)	41 (13)	**< 0.001**
Social support	78 (26)	69 (29)	NS	80 (19)	66 (30)	**0.002**
Dialysis staff encouragement	92 (14)	89 (14)	NS	91 (14)	90 (14)	NS
Patient satisfaction	88 (13)	84 (16)	NS	87 (14)	83 (16)	NS
Kidney disease summary component	62 (10)	52 (8)	**< 0.001**	61 (10)	51 (7)	< **0.001**
**SF-36 components (SD)**						
Physical functioning	68 (28)	30 (29)	**< 0.001**	69 (29)	25 (24)	**< 0.001**
Role-physical	57 (43)	30 (42)	**< 0.001**	61 (42)	24 (39)	**< 0.001**
Pain	78 (23)	56 (30)	**< 0.001**	76 (24)	54 (30)	**< 0.001**
General health	55 (35)	29 (35)	**< 0.001**	58 (35)	23 (32)	**< 0.001**
Emotional well-being	92 (8)	79 (13)	**< 0.001**	90 (9)	79 (13)	**< 0.001**
Social function	68 (20)	52 (25)	**< 0.001**	68 (20)	50 (25)	**< 0.001**
Role-emotional	76 (38)	52 (46)	**< 0.001**	77 (38)	48 (46)	**< 0.001**
Energy/fatigue	65 (15)	38 (23)	**< 0.001**	63 (17)	36 (21)	**< 0.001**
Physical component summary	45 (11)	34 (12)	**< 0.001**	46 (11)	32 (11)	**< 0.001**
Mental component summary	48 (8)	41 (10)	** < 0.001**	47 (8)	40(10)	**< 0.001**

Significant values are in bold.

Data are expressed as mean ± standard deviation for normal data.

*N/S* Not significant.

A multiple linear regression analysis was performed with KDSC, PCS, and MCS as response variables (Table 3). Six explanatory variables were included: age, sex, malnutrition, Barthel Index, and the two main comorbidities: DM and COPD. Malnutrition adjusted for the Barthel Index and COPD showed the highest level of explanation on the KDSC (β = − 8.3, p < 0.001), explaining 28.9% of the variance; the PCS adjusted for the Barthel Index (β = − 11.7, p < 0.001), explaining 33% of the variance; and the MCS adjusted for sex (female) and age (≥ 70 years) (β = − 7.2, p < 0.001), explaining 21.5% of the variance. The comorbidity of DM was not significant predictor of either KDSC, PCS, or MCS (p > 0.05).

**Table 3:** Multiple linear regression according to KDSC, PCS, and MCS variables.

KDSC				PCS			MCS		
	β coefficient	p value	95% CI	β coefficient	p value	95% CI	β coefficient	p value	95% CI
Nutritional status (malnutrition)	− 8.38	**< 0.001**	− 11.87 to 4.89	− 11.77	**< 0.001**	− 16.12 to 7.42	− 7.26	**< 0.001**	− 10.64 to 3.91
Barthel index (independent)	3.08	0.088	− 0.46 to 6.63	6.43	**0.005**	2.02 to 10.84	NA		
COPD (presence)	− 7.44	**0.009**	− 13.0 to − 1.89	NA			NA		
Sex (female)	NA			NA			− 5.73	**0.002**	− 9.24 to − 2.22
Age (≥ 70 years)	NA			NA			3.22	0.061	
DM (presence)	NA			NA			NA		

Significant values are in bold.

Data are expressed as mean ± standard deviation for normal data. *KDSC* Kidney Disease Summary Component, *PCS* physical component summary, *MCS* mental component summary, *NA* not applicable.

In summary, the results of the multiple regression analyses showed that malnutrition was the most prominent predictor of lower scores on the KDQOL-SF among our HD population.

## Discussion

In this cross-sectional study, we observed that the presence of malnutrition was the most consistent independent determinant of decreased health-related QOL in HD patients as assessed by the KDQOL-SF.

Malnutrition and impaired QOL are prevalent conditions among HD patients. Malnutrition can lead to PEW syndrome, which entails a loss of muscle mass and depletion of energy deposits that can cause difficulties in performing the basic activities of daily living and may reduce an individual’s strength and autonomy, which in turn can reduce QOL.

Recommendations and guidelines have been issued for the inclusion of nutritional management in the comprehensive approach to this disease^6^. Thus, it is important to evaluate patients’ nutritional status. Although a gold standard screening technique for detecting malnutrition in HD patients has not been established, higher MIS scores have been associated with poorer nutritional status and higher hospitalization and mortality rates^22^. We evaluated the applicability of the MIS in HD patients and found that 62% of the study population was at NR. These results are in line with the findings of other studies^23,24^, but the percentage of NR found in our work is considerably higher than what has been reported previously^25–27^. However, these prior studies used the SGA screening method. Although SGA is reported to perform well in hospitalized patients, the MIS method was specifically designed to detect the NR in HD outpatients^8^, making it a highly reliable and effective screening tool for assessing NR in this population. The different instruments used may explain the higher rate of NR found in our work.

The presence of NR does not necessary indicate malnutrition, but rather refers to the risk of developing it. In fact, our results demonstrated that 7% of patients at NR were in fact classified as well-nourished. This highlights the importance of implementing a personalized NIP early in order to prevent further deterioration in nutritional status.

It has been demonstrated that MIS correlates with QOL domains as assessed by the KDQOL-SF tool^28^. In our study, after having analyzed the study population’s QOL, we compared it to different variables related to nutritional status. This comparison showed impaired QOL in patients at NR and malnourished patients. Patients with these criteria had lower scores on effects of kidney disease, an area which includes questions about fluid and dietary restrictions. One possible explanation is the fact that a dialysis diet is among the most restrictive diets and these restrictions may cause frustration, represent a significant burden, and lead to suboptimal treatment adherence which in turn may worsen patients' QOL and satisfaction^29,30^.

Many cross-sectional studies have observed poorer QOL outcomes in HD patients compared to well-nourished patients. These studies use different tools to assess nutritional status and QOL^5,31^. A work by Günalay et al.^32^ that used the Mini Nutritional Assessment – Short Form to determine nutritional status demonstrated that QOL scores as measuring by the EQ5D questionnaire decreased as malnutrition rates increased. Uy et al.^33^ studied the relationship between malnutrition and QOL among patients with DM on maintenance HD using the Dialysis Malnutrition Score (DMS) and the World Health Organization Quality of Life (WHOQoL)-BREF questionnaire. They found that those who were malnourished as per the DMS showed a significantly lower physical (p < 0.001), psychological (p < 0.001), and social QOL (p = 0.004).

A prospective research study by Viramontes-Hörner et al.^34^ investigated this association over time and demonstrated that at baseline, malnutrition as assessed by SGA was the only factor independently and negatively associated with QOL, as assessed by the SF-36 and EQ5D questionnaires. They also reported low MCS and PCS scores (< 50) compared to our study population. Several other researchers have also explored the relationship between nutritional status and QOL. They also demonstrated that QOL scores decrease as malnutrition rates increase^3,35^. One strength of our study with respect to previous works is that our work adds to the published data by showing that malnutrition was an independent determinant of decreased QOL on the kidney-specific QOL domains of the KDQOL-SF questionnaire.

The limitations of the study include its cross-sectional design, which prevents causal inference and measurement of associations between nutritional status and QOL over time. Longitudinal studies are needed to clarify the association of nutritional status with QoL. Our working group is currently analysing benefits following the implementation of this nutritional intervention program after one year and determining if there is improvement in both nutritional status and, in turn, quality of life.

## Conclusions

Our study found that malnutrition is one of the most prominent factors affecting QOL. Several previous works have shown the importance of establishing nutritional support through the implementation of a NIP and the use of the MIS as an effective, sensitive screening tool for detecting NR. The high rate of NR observed in our work and the importance of detecting malnutrition in early stages highlight the need to identify at-risk patients early in order to change the timing and type of interventions, personalizing them to improve efficacy. Knowing each patient’s weaknesses on certain areas of the QOL questionnaire allows us to develop and implement new strategies to help patients to improve their general and specific perceptions of QOL. Our findings are supported by other works, but additional studies are needed to further support these assertions.

## Practical application

In this study, we showed that malnutrition is a frequent condition in HD patients and the most powerful factor affecting these patients’ QOL. The use of a reliable screening tool followed by a personalized NIP reduced the risk of developing malnutrition and treatment in early stages improves these patients’ QOL. In view of these findings, early detection of malnutrition and a NIP should be implemented in those who require it. Further long-term observation is needed to assess the potential effects of a NIP on HD patients.
